# Reproductive and metabolic hormone associations in adult Samoan males with and without obesity

**DOI:** 10.1093/emph/eoag001

**Published:** 2026-01-09

**Authors:** Richard G Bribiescas, Kyle S Wiley, Amelia Sancilio, Catherine S Panter-Brick, Satupaitea Viali, Muagututia Sefuiva Reupena, Take Naseri, Nicola L Hawley, Geralyn Messerlian, Stephen T McGarvey

**Affiliations:** Department of Anthropology, Yale University, New Haven, CT 06511, USA; Department of Sociology and Anthropology, University of Texas at El Paso, El Paso, TX, USA; One Vector, Inc, Cambridge, MA 02142, USA; Department of Anthropology, Yale University, New Haven, CT 06511, USA; School of Medicine, National University of Samoa, Apia, Samoa; Lutia I Puava Ae Mapu I Fagalele, Apia, Samoa; Ministry of Health of Samoa, Apia, Samoa; Department of Anthropology, School of Public Health, Yale University, New Haven, CT 06511, USA; Department of Pathology and Laboratory Medicine, Brown University, Providence, RI 02912, USA; Center for Global Public Health, Departments of Epidemiology and of Anthropology, Brown University, Providence, RI 02912, USA

**Keywords:** obesity, follicle stimulating hormone, luteinizing hormone, sex hormone binding globulin, leptin, adiponectin

## Abstract

**Background:**

Obesity is a global health challenge prevalent in Samoa. However, the influence of obesity on adult male reproductive health in Samoa is poorly understood.

**Objectives:**

To determine if reproductive hormone levels differed between adult Samoan males with and without obesity.

**Methodology:**

Reproductive hormones (follicle stimulating hormone [FSH], luteinizing hormone [LH], inhibin b) and sex hormone binding globulin (SHBG) were compared between non-diabetic adult males in Samoa with and without obesity to test the hypothesis that obesity is associated with compromised reproductive function in this population. Metabolic hormones (insulin, leptin, adiponectin), fasting glucose, age, and anthropometrics were assessed and included in multivariable models.

**Results:**

Males with obesity exhibited higher FSH (*P* = 0.002), lower inhibin b (*P* = 0.004) and lower SHBG (*P* < 0.0001). LH levels were similar (*P* = 0.43). Significant associations were evident between LH and FSH (obesity: r^2^ = 0.19, *P* = 0.003; without obesity: r^2^ = 0.24, *P* = 0.001), inhibin b and FSH (obesity: r^2^ = 0.21, *P* = 0.002; without obesity: r^2^ = 0.02, *P* = 0.41), and LH and SHBG (obesity: r^2^ = 0.25, *P* = 0.0005; without obesity: r^2^ 0.01, *P* = 0.49). Multivariable models revealed insulin as an important contributor to inhibin b levels in all males.

**Conclusions:**

Obesity status is an important factor in variation in male reproductive hormone profiles in adult Samoan males.

**Implications:**

Obesity has potentially negative effects on male reproductive hormone function in Samoa. While the effects on male fertility remain unclear, further research is merited.

## BACKGROUND

Obesity is a global health risk with ~10% of the world’s population and one third of North Americans being afflicted as adults [1]. It is associated with metabolic risks such as insulin resistance, type 2 diabetes, hypertension, hyperlipidemia, and cardiovascular disease. Communities undergoing transitions to more imported foods and modern industrial-era diets high in sugars, saturated fats, and simple carbohydrates are particularly vulnerable since these shifts are often accompanied by limited nutrition education, increased sedentism, uneven availability of fresh foods, and other socioeconomic factors [2].

Pacific Islanders show a higher prevalence of obesity and metabolic health challenges compared to other global populations [3]. Using Polynesian-specific thresholds for obesity (Body Mass Index, BMI ≥ 32 kg/m^2^) [4, 5], the prevalence of adult obesity in 2010 was 64.6% among female Samoans and 41.2% among males [6]. Urban Samoans exhibited significantly greater levels of obesity, type 2 diabetes, low High Density Lipoprotein (HDL)-cholesterol, and high triglycerides compared to rural residents [6]. Obesity pervasiveness among Samoans is likely due to genetic and environmental factors that may be unique to this population [7–9]. The introduction of imported food products, high in sugar and fat, and excess consumption of more traditional foods are also contributory to increases in obesity [7, 10].

The impact of obesity on reproductive function in south Pacific populations, including Samoa, is understudied. A limited number of investigations have examined ovarian function in Samoan adult females [11–14]. Studies of male reproductive health in Samoa however are lacking. Health assessments in Samoan and Tongan men in southern California are available but reproductive function was not included [15]. Obesity can compromise hypothalamic–pituitary-testicular (HPT) function and potentially compromise male fertility [16, 17]. A meta-analysis of adult males with various stages of obesity reported higher follicle stimulating hormone (FSH) and estradiol levels as well as reduced inhibin b, testosterone, luteinizing hormone (LH), and sex hormone binding globulin (SHBG) measures [18].

Metabolic hormones such as leptin, adiponectin, and insulin can influence human male reproductive function. Decreases in leptin can attenuate gonadotropin releasing hormone (GnRH) production and downstream production of gonadotropins and sex steroids [19]. However excessive leptin production due to obesity can also lead to declines in HPT function, perhaps via decreases in leptin receptor sensitivity within the hypothalamus [20, 21]. Significant population variation in serum leptin levels has also been observed independent of sex and adiposity [22–24].

Insulin is a peptide hormone produced by the pancreas and is vital for cellular glucose uptake. Insulin receptors are located in the hypothalamus, modulating GnRH, and kisspeptin function, thereby influencing downstream reproductive hormone production [25–27]. A strong and significant association was observed during the manipulation of GnRH levels, insulin sensitivity, and sex steroid production in adult human males. Insulin sensitivity was positively associated with Leydig cell responsiveness to GnRH administration as well as human chorionic gonadotropin (hCG) stimulated increases in testosterone [28].

Adiponectin is a peptide hormone produced primarily within adipocytes. It plays a role in regulating glucose as well as fatty acids and is commonly inversely correlated with circulating leptin levels. Adiponectin tends to be inversely related to testosterone [29, 30], and negatively impacts GnRH production in the hypothalamus [31].

Attention to population variation and the examination of metabolic health challenges under varied ecological conditions can provide insights into the evolutionary biology of obesity as well as the interaction between adiposity and potential effects on reproduction [32]. Moreover, the association between metabolic and reproductive health continues an area of interest in the area of evolutionary medicine [33, 34].

## OBJECTIVES

The objective of this investigation was to increase our understanding of the impact of obesity on reproductive health in adult Samoan males. While the negative effects of obesity on male reproductive health are well documented in many other populations [18], the effects on an under-researched, under-served South Pacific population with a high prevalence of obesity is unknown. Moreover, the association between metabolic hormones associated with obesity with male reproductive function can vary between populations living within different ecologies and lifestyles [23, 24, 35]. We therefore tested the hypothesis that obesity status contributes to variation in adult Samoan male reproductive hormone profiles. Specifically, based on results from clinical studies from other populations, we predicted that FSH, LH, and SHBG levels would be higher in participants with obesity while inhibin b would be lower. We also sought to determine if obesity status co-varied with other contributory factors such as metabolic hormone levels (leptin, adiponectin, insulin), age, anthropometrics, and fasting glucose. Testosterone and estradiol were not measured due to limited sample volumes.

## METHODS

Data was collected in 2010 from participants in the nation of Samoa. The Samoan population in 2010 was estimated at 186 405, life expectancy at birth was 74.2 years, and 38% of the population was <15 years of age [36]. Samoa is considered a lower-middle income economy [37]. Almost 60% of adult males, ≥15 years, were active economically and, 46% of those were formally or informally employed in farming and fishing. Details on the broader investigation have been described earlier [6].

The broader investigation of DNA methylation among 92 Samoan adult males [38], from which the present participants were selected, was a population-based cross-sectional survey of adults aged 24.5–64 years from 33 communities from all census regions, based upon the national population distribution described in the 2006 census [39]. The methylation sample participants were 25–40 years of age, those with obesity had BMI ≥ 32.00 kg/m^2^ and abdominal circumference (AC) ≥ 92.5 cm, and 46 had BMI < 26.00 kg/m^2^ and AC < 92.5 cm) for those without obesity. An AC of 92.5 cm was chosen for classification based on its being the sample median among this age group. The sample of participants (n = 88) for the present study was based on having sufficient stored frozen serum for hormone assays. Sample sizes for some hormone values were < 88 due to serum volume limitations (Table 1).

**Table 1:** Mean comparisons between participants with and without obesity. Differences in sample N's due to missing data.

	Without Obesity				With Obesity					
	N	Median	Mean	SD	N	Median	Mean	SD	*P*	Hedges’ G
Age (years)	42	29.3	30.6	4.9	45	32.9	32.4	4.5	0.08^a^	0.4
Height (cm)	42	173.1	173.2	5.2	45	174.5	174.5	5.1	0.23^a^	0.3
Weight (kg)	42	74.2	73.3	5.6	45	106.6	106.5	9.0	< 0.0001^b^	4.4
BMI (kg/m^2^)	42	24.8	24.4	1.3	45	34.4	35.0	2.5	< 0.0001^b^	5.3
Body Fat %	42	10.2	10.5	3.2	41	31.4	32.1	5.7	< 0.0001^b^	4.7
Abd_Circ (cm)	42	80.4	81.1	3.9	45	106.3	106.8	6.3	< 0.0001^b^	4.9
Hip_Circ (cm)	42	95.6	95.8	3.9	45	113.9	114.2	4.6	< 0.0001^b^	4.3
Tri_Skf (mm)	42	12.5	12.6	4.2	45	21.3	21.1	4.7	< 0.0001^b^	1.9
Leptin (ng/ml)	39	1.9	2.3	1.5	43	9.5	10.0	5.1	< 0.0001^b^	2.0
Adiponectin (μg/ml)	39	5.0	5.5	2.1	43	4.0	3.7	1.1	< 0.0001^b^	1.1
Insulin (mIU/ml)	39	5.0	6.4	5.2	41	11.8	13.9	8.6	< 0.0001^b,c^	1.0
Glucose (mg/dL)	39	81.0	79.3	11.9	43	93.0	95.0	20.6	< 0.0001^b^	0.9
LH (pg/ml)	42	4.7	5.1	2.2	45	4.7	5.2	2.2	0.43^b^	0.05
FSH (IU/L)	41	4.1	4.3	2.3	45	5.7	6.1	3.2	0.002^b^	0.64
SHBG (nmol/L)	42	76.7	79.0	32.7	45	41.5	46.0	27.0	< 0.0001^b^	1.1
Inhibin b (pg/ml)	42	159.5	159.4	56.0	45	123.6	130.8	42.0	0.004^b^	0.58

^a^Two-tailed.

^b^One-tailed.

^c^Insulin log transformed for t test. Descriptive statistics from raw data.

Participants were given detailed written and verbal information in the Samoan language about the study, data collection protocols, and rights during recruitment and provided written informed consent. Research protocols and the informed consent process were approved by the Brown University Institutional Review Board and the Health Research Committee of the Samoan Ministry of Health. Analysis of deidentified serum samples for reproductive hormone analysis was additionally approved by the Yale University Human Subjects Committee.

Anthropometric measures were conducted as described previously [6]. Participants wore light island clothing during the anthropometric exam, height was measured to the nearest 0.1 cm with a portable GPM anthropometer (Pfister Imports Ltd, New York, USA), and weight to the nearest 0.1 kg with a Tanita HD 351 digital weight scale (Tanita Corporation of America, Inc, Illinois, USA). Triceps skinfold was measured with a Lange caliper (Cambridge Scientific Industries, Inc., Maryland, USA), to the nearest 0.1 mm. Abdominal and hip circumferences were measured to the nearest 0.1 cm with a cloth tape. All anthropometry was done in duplicate and averaged for analysis. Bioelectrical impedance (BIA) measures of resistance and reactance were obtained with an RJL BIA-101Q device (RJL Systems, Inc., Michigan, USA) using standard procedures. Fat mass and body fat percentage were calculated using the equations established from direct body composition studies using the dual-energy X-ray absorptiometry (DEXA) method in Polynesians residing in New Zealand [5, 40].

Serum sampling and metabolic biomarker assays were conducted in the following manner. Local phlebotomists collected whole blood specimens after a minimum of 10 h overnight fast. Blood samples were collected in 10 ml vacutainers spray coated with silica and containing polymer gel for serum separation. Samples were centrifuged shortly after collection to separate the serum and then stored at –40^°^C in Samoa before transport on dry ice to Northwest Lipid Labs, Seattle, USA. Leptin, adiponectin, glucose, and insulin were assayed in 2010 at the Northwest Lipid Metabolism and Diabetes Research Laboratories, University of Washington, Seattle. Adiponectin was measured using a latex particle-enhanced turbidimetric immunoassay (Otsuka Pharmaceuticals, Ltd) performed on a Roche Modular P analyzer. The analysis uses latex beads-immobilized with an anti- adiponectin antibody. The assay sensitivity is 0.5 ug/mL and the analytical range is 0.5–25 ug/mL. Inter-assay CVs for high and low levels quality control samples were 1.9% and 2.5%, respectively.

Leptin analysis was performed using a commercial radioimmunoassay kit (EMD Millipore, Inc., St Charles, MO). Assay sensitivity is 0.5 ng/mL. Intra- and inter-assay CVs for the leptin RIA was 3.53% and 5.2%, respectively. Blood glucose was measured enzymatically on a Hitachi 917 Clinical Chemistry auto-analyzer using the glucose hexokinase method [41, 42]. Determination of insulin levels was performed by a two site immuno-enzymometric assay on a TOSOH 2000 autoanalyzer. The assay has a sensitivity level of 0.5 uU/mL. The inter-assay CVs for low, medium, and high level control samples were 2.8%, 2.5%, and 2.0% respectively. Cross-reactivity with Human C-peptide: 0%, intact Proinsulin: 2.0%, Proinsulin split [32, 33]: 2.6%, Proinsulin Des [43, 44]: 39.8%.

Remaining serum was stored at −80^o^C at Brown University after the metabolic biomarker assays were completed. In 2015 reproductive hormone assays were performed in the Yale Reproductive Ecology Laboratory (Yale University, Department of Anthropology) using commercial enzyme immunoassay kits to measure levels of SHBG (11-SHBHU-E01, Alpco, Salem, NH), FSH (11-FSHHU-E01, Alpco), LH (11-LUTHU-E01, Alpco), and inhibin b (A81301, Gen II, Beckman Coulter, Brea, CA), in duplicate, according to manufacturer instructions using a Biotek plate reader (Agilent Technologies, Inc, Santa Clara, CA). When stored at –80^o^C for over 5 years, serum measures of FSH, LH, inhibin b, and SHBG do not exhibit significant degradation [45–47].

Blank wells read below detectable limits. High and low quality control results were within manufacturer performance specifications, with the exception of the low quality control values for SHBG. One inhibin b and one FSH value were excluded from analyses due to read failures. Inter-assay coefficients of variation (CVs) calculated from quality control pools were as follows; SHBG low = 17.7%, high = 13.1; FSH low = 9.7%, high = 5.5; LH low = 19.4%, high = 19.2; inhibin b low = 5.1%, high = 3.0. The mean across assays of the low quality controls for SHBG (49.6 nmol/L) was 6.6% above the recommended manufacturer range (27.3–45.5 nmol/L). Samples were assayed in random order. All reproductive hormone assays were performed by the same co-author (KSW).

### Statistical analyses

Two insulin values were excluded from the analysis due to significant hyperinsulinemia (>50 mUI/L). Remaining insulin values were log transformed prior to statistical analysis due to skewed distribution (Shapiro–Wilk, *P* < 0.0001). Unpaired t-tests assuming unequal variances were used to compare means of hormonal and physiological variables between participants with and without obesity. Unless otherwise indicated, mean comparisons were one-tailed based on *a priori* study design or predictions, such as expected differences in weight between participants with and without obesity. Linear regressions and multivariable linear regressions with obesity status as a nominal variable were conducted based on hypothalamic control of gonadotropins (LH, FSH, inhibin b). Comparison of regression slopes between those with and without obesity, was conducted using analysis of covariance (ANCOVA). When linear regression slopes were not significantly different, associations between metabolic and reproductive hormones were examined using all participants.

Multivariable linear regression was conducted with LH, FSH, SHBG, and inhibin b as dependent variables and anthropometric characteristics and metabolic hormone levels (leptin, adiponectin, insulin) as independent variables. Obesity status (with/without) was entered as a dummy variable. To mitigate collinearity and obtain the most parsimonious models based on AICc values, least absolute shrinkage and selection operator (LASSO) was deployed as the estimation method [48], and variables with Variance Inflation factor (VIF) scores ˃5.0 were excluded from multivariable models to mitigate the risk of over fitting. Due to differing sample sizes and standard deviations between experimental groups, Hedges’ G was used to estimate effect sizes of mean differences and determine the physiological significance of the results. Statistical analyses were conducted using JMP 17.0 (JMP Statistical Discovery LLC, Cary, NC) and Prism 10.0 (GraphPad, Inc., San Diego, CA) both for Macintosh. Alpha was set to 0.05.

## RESULTS

**Figure 1:**
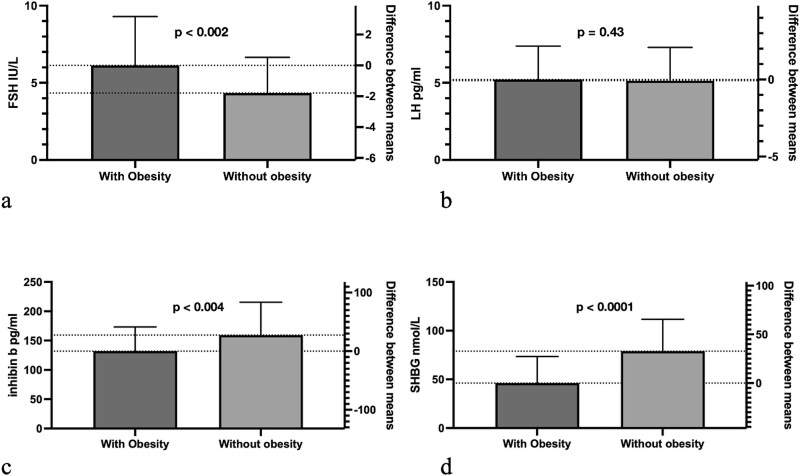
(a)–(d) Mean comparisons of reproductive hormones and SHBG in participants with and without obesity (mean ± sd).

Metabolic hormone and anthropometric measurements exhibited differences that were consistent with obesity status (Table 1). Participants with obesity exhibited significantly higher FSH, lower inhibin b, and lower SHBG levels compared to those without obesity. No significant differences were detected between mean LH levels (Fig. 1d). Males with obesity exhibited higher leptin, insulin, and glucose, as well as lower adiponectin levels compared to those without obesity. Effect sizes were high for anthropometric measures with the exception of height and tricep skinfolds. High leptin, adiponectin, fasting glucose, and insulin Hedges’ G values were consistent with obesity status.

**Figure 2:**
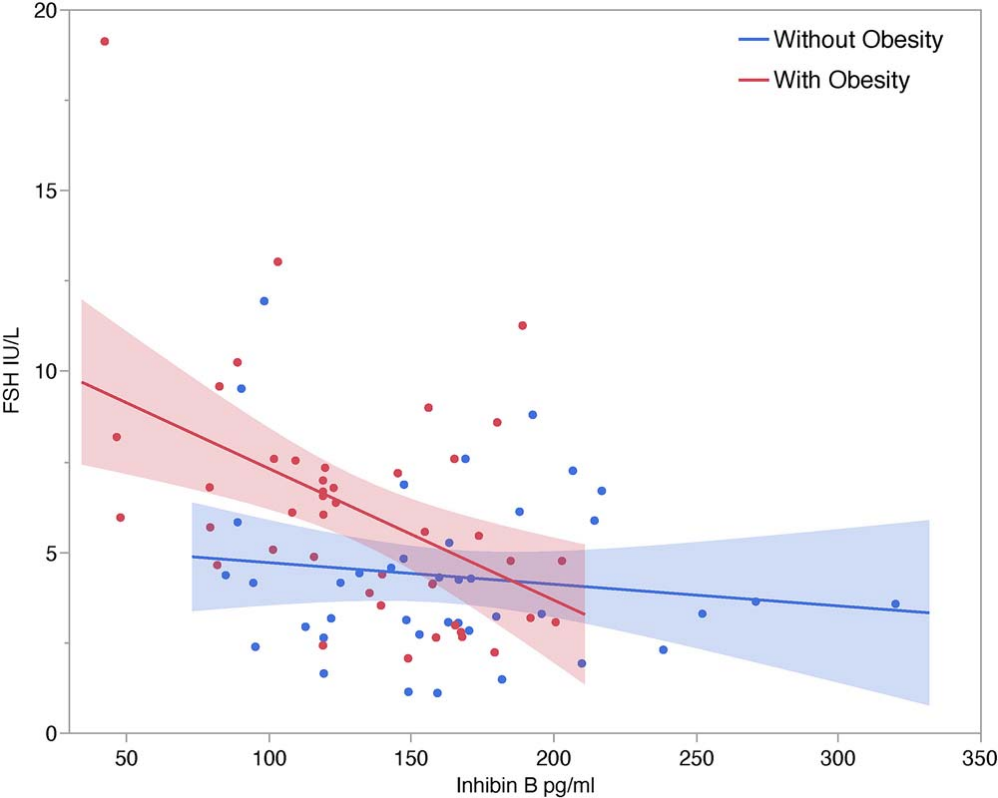
Inhibin b levels (pg/ml) in association with FSH (IU/L) (with obesity r^2^ = 0.21, *P* = 0.002; without obesity r^2^ = 0.02, *P* = 0.41; slope difference ANCOVA *P* = 0.02). Shaded areas indicate 95% confidence intervals.

The linear association between inhibin b and FSH was significant for those with obesity but not for those without obesity. Compared to adult males without obesity, those with obesity exhibited a stronger negative association between inhibin b and FSH (Fig. 2). The slopes and intercepts were significantly different (estimate = 0.01, std error = 0.006, F = 5.33, *P* = 0.02; estimate = 7.99, std error = 0.94, *P* < 0.0001).

**Figure 3:**
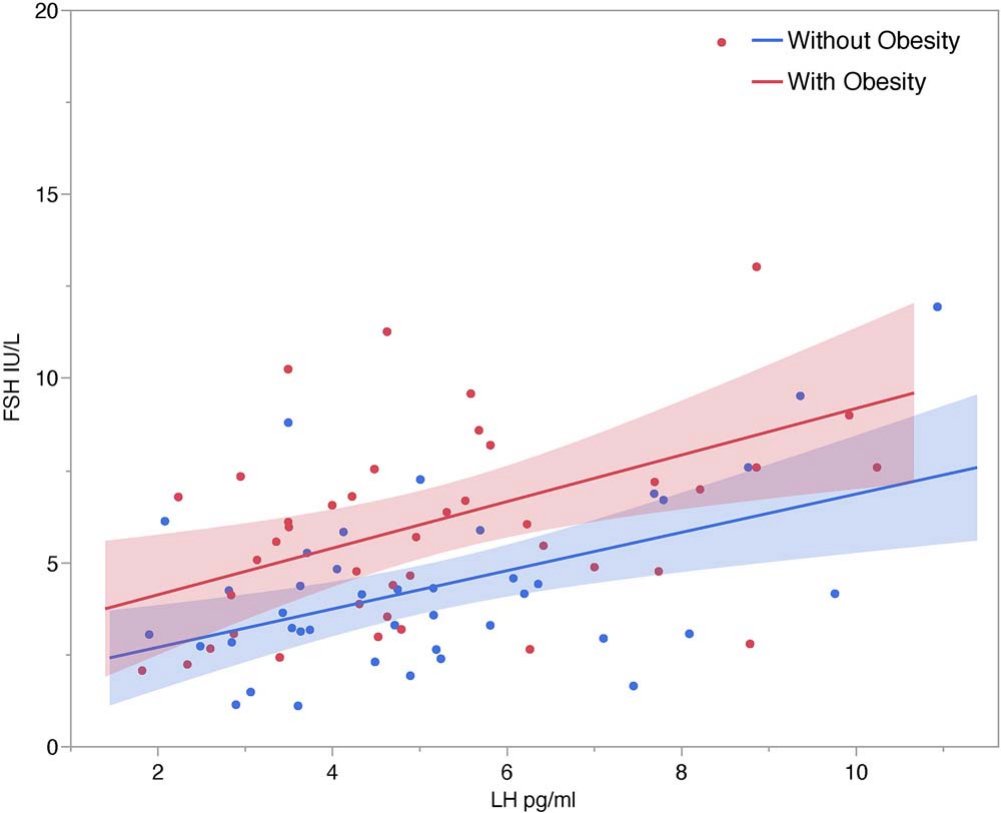
Associations between LH (pg/ml) and FSH (IU/L) values are presented with obesity r^2^ = 0.19, *P* = 0.003; without obesity, r^2^ = 0.24, *P* = 0.001; all r^2^ = 0.19, *P* < 0.0001, slope difference ANCOVA *P* = 0.66.

FSH and LH were positively associated in participants with and without obesity (Fig. 3). The consistent difference and parallel slopes indicate consistently higher FSH levels in adult males with obesity relative to LH variation. The uniformity of the difference is evident with the slopes not being significantly different (estimate = −0.88, std error = 0.27, F = 0.19, *P* = 0.66). The significant difference in the intercepts indicates consistently higher FSH relative to LH (estimate = 2.26, std error = 0.71, *P* < 0.002).

**Figure 4:**
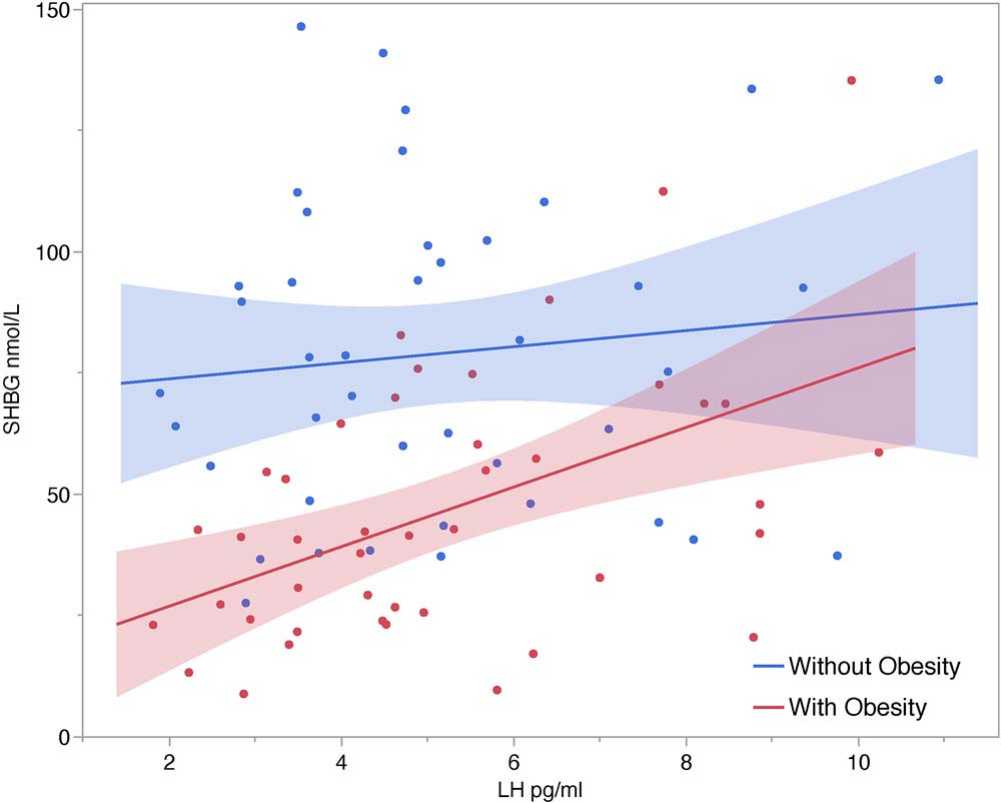
Associations between LH and SHBG in Samoan adult males (with obesity, r^2^ = 0.25, *P* = 0.0005; without obesity, r^2^ = 0.01, *P* = 0.49).

Linear regression associations between SHBG and LH were positive and significant in adult males with obesity although similar associations were not evident in those without obesity (Fig. 4). The slopes (ANCOVA, *P* = 0.03) and intercepts were significantly different indicating a stronger association in males with obesity (ANCOVA, *P* < 0.001). Associations between SHBG and other reproductive hormones were as follows: with obesity FSH r^2^ = 0.03, *P* = 0.23; without obesity r^2^ = 0.11, *P* = 0.04; all r^2^ = 0.002, *P* = 0.68; with obesity inhibin b r^2^ = 0.02, *P* = 0.39; without obesity r^2^ = 0.04, *P* = 0.18; all r^2^ = 0.08, *P* = 0.009).

Associations between reproductive and metabolic hormones are reported in Supplemental Table 1. ANCOVA was conducted when results for both groups combined (All) were significant to determine if regression slopes between those with and without obesity were significantly different. Leptin was modestly associated with inhibin b in males with obesity and with SHBG in all males. Adiponectin was associated with SHBG in males with obesity and all combined, as well as with inhibin b in all males. Insulin was associated with inhibin b in males with obesity and when combined with those without obesity. SHBG was associated with insulin in those with and with obesity and when combined.

Multivariable generalized linear models (individual dependent variables FSH, LH, inhibin b, SHBG) were statistically significant and revealed a predominant contribution of other reproductive hormones regardless of obesity status. With the exception of the association between insulin and inhibin b, metabolic hormones, anthropometric, and obesity status (with/without) had marginal independent contributions on reproductive hormonal variation (Supplemental Table 2).

FSH variation was primarily the result of LH and inhibin b (Supplemental Table 2). Similarly, LH variation was primarily associated with FSH. Inhibin b was the sole dependent variable with a specific significant association with insulin. The model for SHBG did not reveal any independent variable to be significantly influential.

Removing reproductive hormones from each model resulted in less predictive LASSO regressions. The FSH model without other reproductive hormones was (R^2^ = 0.13, *P* < 0.0001, AICc = 387.0) with no non-reproductive hormone independent variable being significant. Removal of reproductive hormones from the LH model resulted in all other variables being removed due to significant collinearity (VIF > 5.0). Similar treatment of Inhibin b model resulted in R^2^ = 0.29, *P* < 0.0001, AICc = 810.1, insulin ß = −0.47, SE = 0.20, *P* = 0.02. SHBG resulted in R^2^ = 0.41, *P* < 0.0001, AICc = 741.9, age ß = 1.49, *P* = 0.04, adiponectin ß = 4.16, *P* = 0.04. Obesity status (with/without) dummy independent variable was only evident in FSH model with other reproductive hormones.

Insulin was the sole metabolic hormone to be a significant contributor to reproductive hormone variation. A post-hoc inhibin b model using linear regression revealed that insulin (log transformed due to non-Gaussian distribution) was negatively correlated with inhibin b in subjects with obesity (r = − 0.37, *P* = 0.01) but not those without (r = −0.17, *P* = 0.29). Multiple linear regression with insulin and obesity status as independent variables resulted R^2^ = 0.16, *P* = 0.001, insulin β = −0.22, *P* = 0.053, obesity status β = 0.26, *P* = 0.02. However, there was notable collinearity in the model.

Simple linear regressions with metabolic hormones (leptin, adiponectin, insulin) as independent variables did not yield significant results for those with or without obesity or when groups were combined (Supplemental Table 3). Similarly, linear regression of reproductive hormones with age as the independent variable is presented in Supplemental Table 4. Luteinizing hormone (LH) and SHBG were significantly associated with age in males with obesity. No associations were found in males without obesity. When all participants were included, FSH exhibited a modest association with age.

## CONCLUSIONS

With the exception of LH, predicted differences in reproductive hormones and SHBG were observed between males with and without obesity. Samoan adult males with obesity exhibited higher FSH and lower inhibin b levels, consistent with potential compromised spermatogenesis. Hormones that control testosterone and estradiol production (LH) did not differ between obese and non-obese groups. Leptin and adiponectin were not associated with reproductive hormones. Insulin was a significant variable contributing to inhibin b variation suggesting that glucose metabolism contributes to variation in hormonal support of spermatogenesis.

FSH in adult Samoan males with obesity was 42% higher compared to those without obesity, suggesting differential GnRH and/or kisspeptin production. FSH producing cells in the pituitary may also be differentially sensitive to GnRH stimulation. Leptin insensitivity by Kiss1 receptors in the hypothalamus of animal models may result in decreased kisspeptin, GnRH, and downstream FSH levels [49]. Since leptin was significantly higher in Samoan males with obesity, this may account for differences in FSH, although there was no association between FSH and leptin. Similarities in LH levels between Samoan males with and without obesity imply that differences in GnRH stimulation are questionable since both LH and FSH are under GnRH control. FSH variation between males with and without obesity is probably due to constrasting inhibin b levels and effects.

FSH levels in Samoan males with obesity were comparable with infertile Egyptian adult males with obesity. However, unlike Samoan male LH results, Egyptian males with infertility and obesity exhibited significantly lower LH levels compared to fertile males with obesity, suggesting differing influences of obesity on hypothalamic function between Samoan and Egyptian males [50]. Fertile adult Australian males with obesity exhibited FSH levels that were ~52% lower and inhibin b levels that were similar compared to Samoan males with obesity in this study [51]. Consequently, the impact of FSH and inhibin b levels on fertility in Samoan males with obesity remains unclear.

The slopes for inhibin b and FSH between Samoan males with and without obesity differ significantly, suggesting a stronger negative feedback effect of inhibin b on FSH in participants with obesity. There is also overlap in the negative feedback function of inhibin b in males with higher inhibin b levels regardless of obesity status. The differences between males with and without obesity lie mainly at the lower range of inhibin b. As inhibin b levels rise, the differences between males with and without obesity diminish.

Higher FSH and lower inhibin b levels are consistent with differences in Sertoli cell function and spermatogenesis. Lower inhibin b levels are associated with Sertoli cell damage or fewer Sertoli cells [50, 52–55]. The negative association between inhibin b and insulin in Samoan men with obesity is consistent with other investigations [56]. While the participants in this investigation were screened for Type II diabetes, higher fasting insulin and glucose in males with obesity indicate higher risk for insulin resistance and potential interference with Sertoli cell function and inhibin b production [17].

No significant differences between obesity groups were noted in LH levels. This contrasts with other reports of reduced LH levels with obesity [57–60]. Our results suggest that LH stimulation of testosterone production is not likely to differ between obese/non-obese groups. Lower testosterone levels in adult males with obesity are often linked to greater aromatization of testosterone into estradiol by adipose tissue [61]. Estradiol can exert a significant negative feedback effect on the hypothalamus, thereby reducing LH and FSH levels and testosterone levels [62]. However, the similarity in LH between Samoan males with and without obesity questions this possibility.

The association between FSH and LH differed between males with and without obesity. While both groups exhibited an expected positive association between FSH and LH given common GnRH regulation, FSH levels per unit of LH were higher in males with obesity. The consistent difference between males with and without obesity suggests distinct negative feedback responses within the HPT axis. Consistently higher FSH levels relative to LH suggest greater FSH stimulation on Sertoli cells in males with obesity [63, 64]. This may be due to a more robust response by the pituitary to produce FSH, and/or decreased inhibitory effects of inhibin b. Given that Samoan males with obesity exhibit significantly lower levels of inhibin b compared to those without obesity, it is probable that inhibin b has a greater effect on FSH levels in males with obesity.

Lower SHBG in adult males with obesity is consistent with other studies [43, 65]. Lower SHBG levels suggest less carrier binding of sex steroids. In contrast to males without obesity, those with obesity exhibited a significant positive association between SHBG and LH. Moreover, SHBG levels overall were lower per unit of LH as well as in general in males with obesity. The relevance of this association is unclear since SHBG is lower on average in Samoan males with obesity. Similar associations between SHBG and LH have also been reported in younger (<50) nondiabetic adult males with obesity [44].

Despite significant differences in leptin, adiponectin, and insulin levels between males with and without obesity, only insulin and inhibin b were correlated. This is partially consistent with other under-served communities that exhibited no associations between leptin and reproductive hormones (FSH, LH, testosterone, estradiol) in healthy non-obese adult males [35].

The limitations of this investigation include the lack of testosterone and estradiol measurements, measures of spermatogenesis, lack of participants over the age of 40, and assessments of conception success. However, the hormones measured in this investigation are among the most informative for understanding male reproductive health [66, 67]. LH and SHBG provide insights into testosterone and estradiol function, although direct measurements would have been optimal. The age of these results merit consideration. Logistical delays were due to the COVID-19 pandemic and other factors. Since 2010 when the serum samples were collected, obesity has increased dramatically among young adult Samoan males (59.6% with BMI ≥30, 2020) meaning that reproductive health may be worsening [68].

The mechanisms by which obesity affects male reproductive hormones are multifaceted, many of which are not addressed in this current study. Obesity-driven increases in inflammation and oxidative stress are evident in Samoa, which can also compromise male reproductive hormone production [69, 70]. Obesity-induced inflammatory responses result in the increased expression of macrophages and cytokines that may negatively affect hypothalamic function and downstream reproductive hormones [57]. These and other factors merit attention in future investigations of male Samoan reproductive function.

## IMPLICATIONS

Obesity appears to have a negative impact on Samoan male reproductive health. Underserved populations that have been subjected to unhealthy changes in diet are likely to be more vulnerable to the harmful impacts of obesity on male reproductive health. This study highlights the need to extend research beyond clinical/urban settings that are commonly the focus of male reproductive and metabolic health research. Additional efforts should be made to promote reproductive, metabolic, and dietary communal educational engagement with the Samoan male population.

## Supplementary Material

eoag001_Supplemental_Files

## Data Availability

Data from the larger study are available in dbGaP [dbGaP ID phs000914]. Hormone data specific to this analysis are available upon reasonable request. Review of the request by the Samoa Ministry of Health's Health Research Committee will be needed. Please contact Dr. Nicola Hawley (nicola.hawley@yale.edu) for further information about this process.
