# Supplementary material for: Reproductive and metabolic hormone associations in adult Samoan males with and without obesity
Source: Evol Med Public Health. 2026 Jan 9;14(1):eoag001. doi: 10.1093/emph/eoag001 (PMC13014357; doi:10.1093/emph/eoag001)
Supplement: eoag001_Supplemental_Files [file eoag001_supplemental_files.zip › Running_Glossary_EMPH_Samoa_Males_revision_eoag001.docx]

**Running Glossary**

**Estradiol**: A steroid hormone produced by testes and adipose tissue in males and by the ovaries in females. It is produced at lower levels in males compared to females and involved with negative feedback loop to hypothalamus. Estradiol tends to be higher in adult males with obesity due to the conversion of testosterone by the enzyme aromatase in adipose tissue.

**Testosterone**: A steroid hormone that is produced primarily in the Leydig cells of the testes in response to luteinizing hormone (LH). It is produced in lower quantities in females by the ovaries. Testosterone supports sexually dimorphic anabolic tissue growth and maintenance, spermatogenesis, secondary sexual characteristics, and libido.

**Gonadotropin Releasing Hormone (GnRH)**: A protein hormone produced within the hypothalamus in both males and females. It regulates and supports the production and pulsatile secretion of gonadotropins (FSH and LH) by the pituitary gland.

**Follicle Stimulating Hormone (FSH)**: A protein hormone produced within the pituitary gland in response to GnRH stimulation. It supports gamete production in both males and females. In males, FSH binds to receptors on the Sertoli cells within the testes and promotes spermatogenesis. FSH is commonly inversely associated with inhibin b.

**Luteinizing Hormone (LH)**: A protein hormone produced within the pituitary gland in response to GnRH stimulation in both females and males. In males, LH binds to receptors within the Leydig cells within the testes and promotes the production of testosterone and to a lesser extent, estradiol.

**Inhibin b**: A protein hormone that promotes gamete production in both females and males. In males, it is produced within the testes, is positively associated with spermatogenesis, and has an inhibitory effect on FSH production.

**Sex Hormone Binding Globulin (SHBG)**: A carrier binding protein for sex steroids such as testosterone and estradiol. It is produced in the liver, binds to sex steroids, and regulates the availability and ability of testosterone and estradiol to bind and activate target receptors. Higher levels of SHBG tends to result in greater binding and decreases in sex steroid activity.

**Leptin**: A protein hormone that is produced primarily by adipose tissue cells (adipocytes). It is commonly positively associated with adiposity and serves as a signal protein to the brain for available energy stores in the form of fat. It can have effects on satiety and reproductive function but there are significant differences between males and females and between populations.

**Adiponectin**: A protein hormone that is produced within adipose tissue and is commonly inversely related to leptin. It is associated with satiety, glucose regulation, and fatty acid oxidation.

**Insulin**: A protein hormone that is produced by the pancreas and is central for glucose metabolism. High levels are common in individuals with obesity, often indicative of insulin insensitivity, and type II diabetes.

**Kisspeptin**: A protein hormone that is produced within the preoptic area (POA), infundibular nucleus, and hypothalamus. It regulates and supports GnRH production.
