# Supplementary material for: Reproductive and metabolic hormone associations in adult Samoan males with and without obesity
Source: Evol Med Public Health. 2026 Jan 9;14(1):eoag001. doi: 10.1093/emph/eoag001 (PMC13014357; doi:10.1093/emph/eoag001)
Supplement: eoag001_Supplemental_Files [file eoag001_supplemental_files.zip › Supplementary_Table_1_EMPH_Samoa_Males_revision_eoag001.docx]

| **Leptin** | **With Obesity** r^2^, p, *ANCOVA p* | **Without Obesity** | **All** |
| --- | --- | --- | --- |
| FSH | 0.02, 0.42 | 0.03, 0.29 | 0.08, 0.009 |
| LH | 0.008, 0.57 | 0.006, 0.64 | 9.45e-5, 0.93 |
| Inhibin b | 0.0003, 0.91 | **0.10, 0.05** | 0.04, 0.07 |
| SHBG | 0.04, 0.22 | 0.02, 0.44 | **0.18, < 0.0001,** *0.59* |
|  |  |  |  |
| **Adiponectin** |  |  |  |
| FSH | 0.01, 0.44 | 5.24e-6, 0.99 | 0.01, 0.28, 0.43 |
| LH | 0.02, 0.39 | 0.002, 0.77 | 0.001, 0.75 |
| Inhibin b | 0.04, 0.18 | 0.04, 0.23 | **0.11, 0.003,** *0.67* |
| SHBG | **0.09, 0.05** | 0.08, 0.07 | **0.21, < 0.001,** *0.53* |
|  |  |  |  |
| **Insulin** |  |  |  |
| FSH | 0.0001, 0.95 | 0.008, 0.58 | 0.02, 0.16 |
| LH | 0.05, 0.18 | 0.009, 0.57 | 0.008, 0.42 |
| Inhibin b | **0.15, 0.01** | 0.03, 0.29 | **0.15, 0.003,** *0.49* |
| SHBG | **0.23, 0.002** | **0.12, 0.03** | **0.32, <0.0001**,*0.74* |

Supplementary Table 1: Individual linear regressions between reproductive and metabolic hormones (r^2^, regression p, ANCOVA p [italics]when necessary to illustrate differences in slopes).
