# Supplementary material for: Reproductive and metabolic hormone associations in adult Samoan males with and without obesity
Source: Evol Med Public Health. 2026 Jan 9;14(1):eoag001. doi: 10.1093/emph/eoag001 (PMC13014357; doi:10.1093/emph/eoag001)
Supplement: eoag001_Supplemental_Files [file eoag001_supplemental_files.zip › Supplementary_Table_2_EMPH_Samoa_Males_revision_eoag001.docx]

| **Dependent**  *Independent* | Beta | STD Error | LASSO R^2^ | p | AICc  LASSO/Standard Least Squares |
| --- | --- | --- | --- | --- | --- |
| **FSH** |  |  | **0.40** | **< 0.0001** | 398.7/421.3 |
| *LH* | 0.40 | 0.13 |  | **0.003** |  |
| *Inhibin b* | - 0.02 | 0.01 |  | **0.02** |  |
| *SHBG* | 0.02 | 0.01 |  | **0.05** |  |
| *Intercept* | 5.03 | 2.73 |  | 0.07 |  |
| *Insulin* | - 0.02 | 0.01 |  | 0.09 |  |
| *Leptin* | 0.15 | 0.10 |  | 0.15 |  |
| *Tricep Skinfold* | - 0.09 | 0.08 |  | 0.24 |  |
| *With/Without Obesity* | 1.12 | 1.23 |  | 0.35 |  |
| *Glucose* | - 0.01 | 0.02 |  | 0.49 |  |
| *Percent Body Fat* | 0.03 | 0.06 |  | 0.68 |  |
| *Age* | 0.01 | 0.07 |  | 0.84 |  |
|  |  |  |  |  |  |
| **LH** |  |  | **0.28** | **< 0.0001** | 329.3/357.1 |
| *FSH* | 0.23 | 0.08 |  | **0.004** |  |
| *Intercept* | 14.42 | 9.49 |  | 0.13 |  |
| *SHBG* | 0.01 | 0.01 |  | 0.13 |  |
| *Height* | - 0.06 | 0.05 |  | 0.20 |  |
| *Inhibin b* | - 0.004 | 0.005 |  | 0.38 |  |
| *Glucose* | 0.01 | 0.02 |  | 0.63 |  |
|  |  |  |  |  |  |
| **Inhibin b** |  |  | **0.40** | **< 0.0001** | 804.5/812.4 |
| *Insulin* | - 0.54 | 0.16 |  | **0.001** |  |
| *FSH* | - 4.37 | 1.53 |  | **0.004** |  |
| *Intercept* | 307.5 | 225.9 |  | 0.17 |  |
| *Leptin* | 1.57 | 1.38 |  | 0.26 |  |
| *Weight* | - 0.46 | 0.43 |  | 0.29 |  |
| *Glucose* | - 0.22 | 0.21 |  | 0.30 |  |
| *SHBG* | 0.23 | 0.23 |  | 0.32 |  |
| *LH* | - 2.11 | 2.23 |  | 0.34 |  |
| *Adiponectin* | 3.01 | 5.48 |  | 0.58 |  |
| *Height* | - 0.57 | 1.27 |  | 0.65 |  |
|  |  |  |  |  |  |
| **SHBG** |  |  | **0.46** | **< 0.0001** | 744.8/759.5 |
| *Adiponectin* | 3.74 | 2.04 |  | 0.07 |  |
| *Age* | 1.33 | 0.79 |  | 0.07 |  |
| *Leptin* | - 1.02 | 0.88 |  | 0.25 |  |
| *LH* | 1.86 | 1.77 |  | 0.29 |  |
| *Inhibin b* | 0.07 | 0.10 |  | 0.45 |  |
| *Glucose* | - 0.11 | 0.15 |  | 0.46 |  |
| *FSH* | 0.78 | 1.12 |  | 0.49 |  |
| *Percent Body Fat* | - 0.52 | 0.91 |  | 0.57 |  |
| *Intercept* | 25.30 | 78.45 |  | 0.75 |  |
| *Hip Circumference* | - 0.18 | 0.86 |  | 0.83 |  |

Supplementary Table 2: Generalized linear regression with LASSO of all participants with obesity status (with/without obesity) included as a dummy variable. AICc for LASSO generalized and standard least squares models reported to demonstrate improvement of model with use of LASSO. Independent variables excluded by LASSO due to excessive collinearity (VIF > 5.0) are not shown.
