# Supplementary material for: Reproductive and metabolic hormone associations in adult Samoan males with and without obesity
Source: Evol Med Public Health. 2026 Jan 9;14(1):eoag001. doi: 10.1093/emph/eoag001 (PMC13014357; doi:10.1093/emph/eoag001)
Supplement: eoag001_Supplemental_Files [file eoag001_supplemental_files.zip › Supplementary_Table_3_EMPH_Samoa_Males_revision_eoag001.docx]

| *With Obesity* | R^2^ | p | AICc |
| --- | --- | --- | --- |
| **FSH** | 0.03 | 0.80 | 224.00 |
| **LH** | 0.03 | 0.72 | 198.52 |
| **Inhibin b** | 0.20 | **0.04** | 423.89 |
| **SHBG** | 0.24 | 0.02 | 388.85 |
| *Without Obesity* |  |  |  |
| **FSH** | 0.04 | 0.70 | 182.13 |
| **LH** | 0.02 | 0.89 | 179.66 |
| **Inhibin b** | 0.14 | 0.14 | 422.25 |
| **SHBG** | 0.17 | 0.08 | 388.60 |

Supplementary Table 3: Multiple linear regression metabolic hormone model (leptin, adiponectin, insulin) as independent variables and individual reproductive hormones as dependent variables. All VIF values of independent variables were < 5.0. Model of inhibin b in males with obesity included significant contribution of insulin as an independent variable (p = 0.01).
