# Supplementary material for: Reproductive and metabolic hormone associations in adult Samoan males with and without obesity
Source: Evol Med Public Health. 2026 Jan 9;14(1):eoag001. doi: 10.1093/emph/eoag001 (PMC13014357; doi:10.1093/emph/eoag001)
Supplement: eoag001_Supplemental_Files [file eoag001_supplemental_files.zip › Supplementary_Table_4_EMPH_Samoa_Males_revision_eoag001.docx]

| *With Obesity* | r^2^ | p |
| --- | --- | --- |
| **FSH** | 0.01 | 0.52 |
| **LH** | 0.001 | 0.86 |
| **Inhibin b** | 0.01 | 0.47 |
| **SHBG** | 0.05 | 0.17 |
| *Without Obesity* |  |  |
| **FSH** | 0.07 | 0.08 |
| **LH** | **0.12** | **0.03** |
| **Inhibin b** | <0.001 | 0.98 |
| **SHBG** | **0.22** | **0.002** |
| *All* |  | **0.66 ANCOVA** |
| **FSH** | **0.05** | **0.04** |
| **LH** | 0.04 | 0.08 |
| **Inhibin b** | < 0.001 | 0.98 |
| **SHBG** | 0.04 | 0.05 |

Supplementary Table 4: Simple linear regression of reproductive hormones with age as the independent variable.
